# The Susceptibility of BALB/c Mice to a Mouse-Adapted Ebola Virus Intravaginal Infection

**DOI:** 10.3390/v15071590

**Published:** 2023-07-21

**Authors:** Olivier Escaffre, Terry L. Juelich, Jennifer K. Smith, Lihong Zhang, Nigel Bourne, Alexander N. Freiberg

**Affiliations:** 1 Department of Pathology, University of Texas Medical Branch, Galveston, TX 77555-0609, USA; 2 Institute for Human Infections & Immunity and Sealy & Smith Foundation, University of Texas Medical Branch, Galveston, TX 77555-0609, USA; 3 Department of Microbiology and Immunology, University of Texas Medical Branch, Galveston, TX 77555-0609, USA; 4 Department of Pediatrics, University of Texas Medical Branch, Galveston, TX 77555-0609, USA; 5 Sealy Institute for Vaccine Sciences, University of Texas Medical Branch, Galveston, TX 77555-0609, USA; 6 Center for Biodefense and Emerging Infectious Diseases, University of Texas Medical Branch, Galveston, TX 77555-0609, USA

**Keywords:** Ebola virus, sexual transmission, intravaginal infection, BALB/c mice

## Abstract

Ebola virus (EBOV) causes Ebola virus disease (EVD), which is characterized by hemorrhagic fever with high mortality rates in humans. EBOV sexual transmission has been a concern since the 2014–2016 outbreak in Africa, as persistent infection in the testis and transmission to women was demonstrated. The only study related to establishing an intravaginal small animal infection model was recently documented in IFNAR^−/−^ mice using wild-type and mouse-adapted EBOV (maEBOV), and resulted in 80% mortality, supporting epidemiological data. However, this route of transmission is still poorly understood in women, and the resulting EVD from it is understudied. Here, we contribute to this field of research by providing data from immunocompetent BALB/c mice. We demonstrate that progesterone priming increased the likelihood of maEBOV vaginal infection and of exhibiting the symptoms of disease and seroconversion. However, our data suggest subclinical infection, regardless of the infective dose. We conclude that maEBOV can infect BALB/c mice through vaginal inoculation, but that this route of infection causes significantly less disease compared to intraperitoneal injection at a similar dose, which is consistent with previous studies using other peripheral routes of inoculation in that animal model. Our data are inconsistent with the disease severity described in female patients, therefore suggesting that BALB/c mice are unsuitable for modeling typical EVD following vaginal challenge with maEBOV. Further studies are required to determine the mechanisms by which EVD is attenuated in BALB/c mice, using maEBOV via the vaginal route, as in our experimental set-up.

## 1. Introduction

Ebola diseases (EBOD) are caused by several single-stranded negative sense RNA virus species, including *Sudan ebolavirus* and *Zaire ebolavirus*, that are members of the *Filoviridae* family and *Ebolavirus* genus. EBOD are characterized in humans by a febrile illness with diffuse internal and external bleeding and often leads to high mortality rates, depending on the size of the outbreak [1,2]. Licensed vaccines have been available since 2019 [3] but outbreaks still occur with the most recent ones being in 2022–2023 in Uganda and Democratic Republic of the Congo [4] where there were 164 (34% fatality) and 6 confirmed cases (100% fatality) of, respectively Sudan virus disease (SVD) or Ebola virus disease (EVD).

EVD is caused by Ebola virus (EBOV), which has been the most studied ebolavirus species since its discovery in 1976 [5]. However, sexual transmission is either rare, or has been under-reported compared to other modes of transmission, as it was only documented during the large 2014–2016 EBOV outbreak [6,7]. This is despite knowing that virus has been found in the testes of non-human primates since 1985 [8], and knowing since 1968 that another closely related filovirus (Marburg virus) can be sexually transmitted [9]. Indeed, while it is generally observed that most outbreaks result from spillover events, others, including sporadic flare-ups, are suspected to have emerged because of sexual transmission from male survivors [10,11,12,13,14,15] that can harbor the virus genome in their testes for an extended period of time. Such events were documented in 1995 [16] and then extensively during the 2014–2016 outbreak in Africa [17,18,19,20,21,22,23,24]. A mathematical study even suggested that isolating infectious individuals and having them avoid sexual intercourse were efficient ways to end the 2014 outbreak [25], altogether highlighting the impact of the sexual route as potentially significant in EBOV transmission. Yet, this route is still poorly understood for all filoviruses.

In an attempt to fill the gap in knowledge regarding EBOV sexual transmission in women, a recent study documented the successful intravaginal infection of mice deficient for type I interferon receptors (IFNAR^−/−^), using a mouse-adapted (maEBOV) or wild-type EBOV isolate [26]. Most of the subjects succumbed to disease by Day 9 post infection, similar to results observed using intraperitoneal injection with the same dose [27]. Histopathological analysis of the uterus showed macrophage infiltrations in the muscular outer layer, as well as the presence of EBOV antigen within the epithelial layer. Consistent with these results, and while there are no specific data on mouse vaginal tissues from that study, we previously demonstrated in vitro that the human vaginal epithelium cultured at the air–liquid interface is susceptible to EBOV infection and mounts a robust inflammatory response consistent with vaginitis, with increased secretion of IL-6, IL-8, and IP-10 [28,29]. Overall, EBOV pathogenesis in the female reproductive tract is still poorly understood, and there is a need to develop additional animal models mimicking EBOV sexual transmission in women and characterizing in vivo vaginal and uterine infection, in order to develop countermeasures (other than condoms) to prevent virus transmission.

Here, we sought to characterize EBOV sexual transmission in wild-type immunocompetent BALB/c mice following human-assisted intravaginal inoculation of maEBOV, which is known to cause distinct mortality rates, depending on the route of infection used in this animal model.

## 2. Materials and Methods

Ethics statement: Animal experiments were approved by the Institutional Animal Care and Use Committee of The University of Texas Medical Branch (UTMB) (IACUC protocol 2104028), and performed following the guidelines of the Association for Assessment and Accreditation of Laboratory Animal Care International (AAALAC) by certified staff in an AAALAC-approved facility.

Virus and cells: Mouse-adapted Ebola virus (maEBOV) (originally termed EBO-Z ’76 Mp3 Vp2 Mp9 GH, derived from the 1976 Mayinga isolate through serial passages in mice and double-plaque purification [30]) was passaged twice in Vero E6 cells (ATCC, CRL1586) at UTMB before purification via ultracentrifugation on a 20% sucrose cushion. A virus pellet was then resuspended in fresh Dulbecco’s modified Eagle’s medium (DMEM) with 5% fetal bovine serum (FBS) to make a concentrated working stock. Virus titration was performed in Vero E6 cells via a plaque assay, and expressed as log10 (pfu/mL), as previously described [31]. However, one should note that titration results were converted and expressed at log10 PFU/gram when quantifying the virus in tissues. All cell cultures tested negative for mycoplasma contamination. All infectious work was carried out under biosafety level 4 conditions at UTMB.

Study design: Briefly, 12-to 16-week-old female BALB/cAnNHsd (BALB/c) mice were purchased from Envigo (Somerset, NJ, USA). When appropriate for the study, anesthetized mice received a single dose of long-acting medroxyprogesterone acetate (3 mg/mouse of Depo Provera^®^) via subcutaneous injection in the scruff of the neck 6 days prior to virus challenge. A first vaginal swab was performed on each anesthetized animal prior to virus challenge with a PBS wet sterile applicator that was gently rolled against the vaginal wall to clear the vaginal cavity. The virus (15 μL virus in PBS) was then immediately inoculated intra-vaginally (I.V.) or intraperitoneally as a control (I.P.) (Day 0). The delivered dose was verified with a plaque assay technique started on the same day. Mice were monitored daily for 21 days for development of clinical signs of disease and weight loss. The health condition of each animal was scored as follows: 1 for healthy, 2 for lethargic with ruffled fur, 3 for scoring 2 plus hunched posture and orbital tightening, and 4 when ≥20% weight loss or for scoring 3 plus reluctance to move when stimulated, showing paralysis, unable to access food/water, or displaying a moribund appearance. Vaginal swabs were also collected every other day to detect active virus replication starting on Day 2, and specimens were subsequently stored frozen in 250 μL PBS/1% bovine serum albumin. Blood was collected at time of euthanasia.

The susceptibility study comprised three groups of six mice. A positive control group received progesterone pretreatment and was inoculated with 10^4^ plaque-forming units of virus (PFU) by I.P. route. The two other groups received the same dose administered I.V. with or without progesterone pretreatment. The serial dose study was performed to determine the mean lethal dose (LD_50_),and comprised five groups (N = 10 each) of progesterone-primed mice with a virus doses ranging from 10^3^ to 10^7^ PFU via the I.V. route. Genital tract tissues (from the vagina, cervix, uterine horns, and ovaries), kidney, liver, spleen, and blood were collected at time of euthanasia. Tissues were then homogenized in PBS for virus detection via a plaque assay as previously described [28].

Plaque neutralization assay: Vero E6 cells were seeded in 12-well plates and cultured in DMEM 10% FBS until full confluency. Heat-inactivated sera from naïve and maEBOV-infected mice collected at the time of required euthanasia or the end of study (Day 21) were 2-fold serially diluted in DMEM 2% FBS for testing. The virus was diluted in the same media to yield 80 plaques/100 μL, and a 1:1 volume was added to the diluted sera for 1 h at 37 °C/5% CO_2_. The mixture (200 μL/well) was then transferred to cells for another hour at 37 °C/5% CO_2_, with gentle rocking every 15 min. The overlay (tragacanth gum/MEM mixture with 2% FBS) was then added in a manner similar to a plaque assay [31]. The neutralization titer (PRNT_50_) of the serum was defined as its reciprocal dilution in which the virus plaque count is reduced by 50% when compared with the corresponding count from the virus control. PRNT_50_ was calculated using normalized data on a variable slope model.

Statistical analysis: The Kaplan–Meier method was used to represent survival estimation, and a log-rank test was then performed for the comparison of survival curves (** *p* < 0.01). Pearson’s correlation was performed to determine whether disease severity and seroconversion vary together. Comparison of viral load detected from the vaginal swabs between groups and days was assessed by an ANOVA, followed by Dunnett’s multiples comparison test (* *p* < 0.05, ** *p* < 0.01, *** *p* < 0.001).

## 3. Results

### 3.1. The Susceptibility of BALB/c Mice to maEBOV Intravaginal Challenge

**Figure 1:**
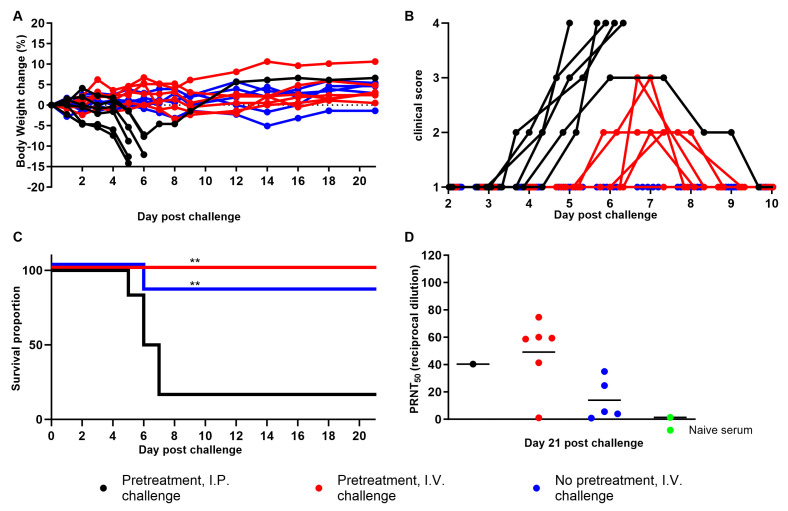
Susceptibility of BALB/c mice to maEBOV by intraperitoneal (I.P.) and intravaginal (I.V.) challenge. Mice (N = 6/group) were challenged with a targeted dose of 10^4^ pfu and monitored for up to 21 days for weight loss (**A**), clinical signs of disease (**B**), and survival (**C**). Seroconversion to infection was assessed on the last day with a plaque neutralization assay, and PRNT_50_ (reciprocal dilution) was calculated (**D**). Black, red, and blue symbols (●, ●, ●)/lines represent subjects from the I.P. challenge (progesterone-primed), I.V. challenge (progesterone-primed), or I.V. challenge (no pretreatment) group, respectively. Green symbol (●) represents data from the serum of a naïve animal. Double asterisks (**) indicate statistical differences of *p* < 0.01 for the mortality rate when compared to mice from the I.P. challenge control group.

We first sought to determine whether wild-type mice were susceptible to maEBOV using the vaginal route. When applicable, progesterone treatment was used prior to virus challenge to synchronize the estrous cycle to the diestrus stage [32] and to thin the vaginal epithelium in order to increase the likelihood of infection. Groups of six mice received a target dose of 10^4^ PFU of virus per subject by either the I.P. or I.V. route. The effective delivered dose was 1.93 and 1.85 × 10^4^ PFU per animal using the I.P. or I.V. route, respectively. As expected in the control group that received progesterone pretreatment and I.P. challenge, mice started losing weight as early as Day 1 (Figure 1A), showing other clinical signs of disease at Day 4 (Figure 1B), and five of them succumbed to disease by Day 6 (Figure 1C). The remaining animal scored 3 until Day 7, but fully recovered by Day 10, and seroconverted to a PRNT_50_ titer of 40 (reciprocal dilution) by Day 21 (Figure 1D).

**Figure 2:**
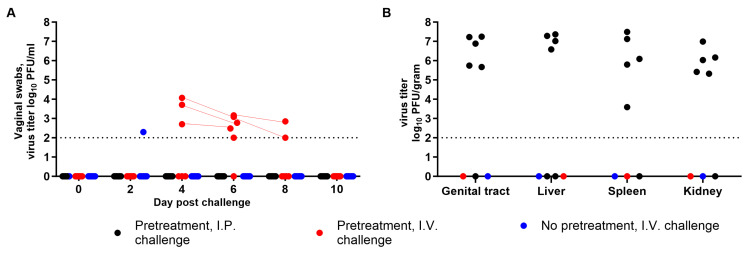
Detection of infectious virus in swabs and tissues from BALB/c mice following maEBOV intraperitoneal (I.P.) and intravaginal (I.V.) challenge in a susceptibility study. Detection of virus replication in the vaginal vault from swabs collected every other day until Day 21 (**A**), and the presence of virus in tissues from subjects that succumbed to disease or after euthanasia at Day 21 (**B**). Black, red, and blue symbols (●, ●, ●) represent subjects from the I.P. challenge (progesterone pretreatment), I.V. challenge (progesterone pretreatment), or I.V. challenge (no pretreatment) group, respectively. Dotted line represents the limit of detection of 100 PFU/mL.

Interestingly, no animals from the two intravaginal challenge groups required euthanasia or succumbed to disease (*p* ≤ 0.01) (Figure 1C). Note that one mouse from the untreated group did not recover from anesthesia during vaginal swabbing on Day 6 (Figure 1C). Specifically, among the six animals that received progesterone pretreatment, only one transiently lost a significant amount of weight (8%) from Day 6 to 8 (Figure 1A), but all of them exhibited mild clinical signs of disease during that time (Figure 1B), and five seroconverted to a PRNT_50_ titer higher than the positive control, ranging from 41 to 75 (Figure 1D). In the no progesterone group, one mouse transiently lost 5% of its initial body weight (Figure 1A), while two others seroconverted to a PRNT_50_ titer of 25 and 35 (Figure 1D). Note that PRNT_50_ titers from the three other subjects were under 5, and not calculable. A positive correlation was established between displaying clinical signs of disease and higher PRNT_50_ titer after an I.V. challenge (Pearson r = 0.73; *p* ≤ 0.01). Altogether, these results suggest that progesterone priming increased susceptibility to infection, and the chance of developing symptoms of disease as well as induced higher antibody levels. In addition, the virus was detected in the vaginal vault of three progesterone-treated mice starting at Day 4, in five at Day 6, and in only two by Day 8 post I.V. challenge (Figure 2A), consistent with lasting shedding of virus from the human vaginal epithelium [29]. Note that infectious virus was never found in swabs from one subject in that group, despite detecting clinical signs of disease and seroconversion. In contrast, in the non-progesterone group, the virus was detected in the vaginal vault of only one mouse, and only on Day 2. This sporadic occurrence could be explained as the detection of residual inoculum instead. Interestingly, infectious virus was never isolated from the vaginal swabs of any animal in the I.P. control group (with a limit of detection (LOD) of 100 PFU/mL), and only animals that succumbed to disease (83% of control group) had infectious virus in their tissues, which included reproductive tract tissues likely due to viremia (Figure 2B) and is consistent with previous studies in guinea pigs and rhesus macaques using the I.P. route [33,34,35]. However, this also suggests that female mice infected via the I.P. route and presenting with EVD symptoms would not transmit the virus via the sexual route. Although these results may be virus dose- and estrous cycle-dependent, the data suggest that maEBOV can infect wild-type BALB/c mice through vaginal inoculation, wherein it can actively replicate for at least 8 days. However, overall, this route of infection causes significantly less disease compared to the I.P. route at a similar dose of the same virus stock.

### 3.2. The Virus Dose Response of BALB/c Mice to maEBOV Intravaginal Challenge

**Figure 3:**
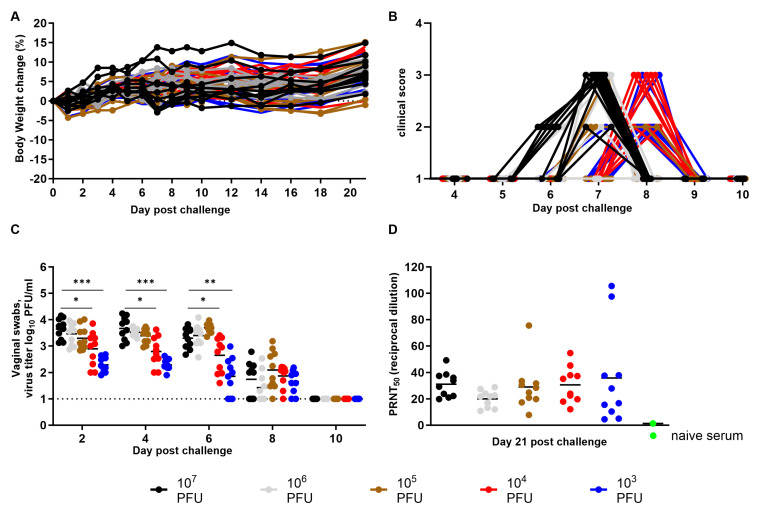
Serial dose study of progesterone-primed BALB/c mice following maEBOV intravaginal challenge. Mice (N = 10/group) were challenged with a targeted dose of 10^3^ to 10^7^ PFU, and monitored for up to 21 days for weight loss (**A**) and clinical signs of disease (**B**). Detection of virus replication in the vaginal vault from swabs collected every other day until day 21 (**C**). Dotted line represents the limit of detection of 10 PFU/mL. Seroconversion to infection was assessed on the last day using a plaque neutralization assay and PRNT_50_ (reciprocal dilution) was calculated (**D**). Black, gray, brown, red, and blue symbols (●, ●, ●, ●, ●) represent subjects from the 10^7^, 10^6^, 10^5^, 10^4^, and 10^3^ PFU group, respectively. Green symbol (●) represents data from the serum of a naïve animal. Asterisks indicate statistical differences of *p* < 0.05 (*), *p* < 0.01 (**), and *p* <0.001 (***) for average virus titer between the 10^7^ and 10^3^ or 10^4^ groups.

In view of the results from the susceptibility study and as a proof of concept, progesterone priming was subsequently used for all mice in the serial dose study to determine the dose causing 50% lethality (LD_50_). Five groups of ten mice received a target dose ranging from 10^3^ to 10^7^ PFU of virus per subject using the I.V. route. The effective delivered dose was 5.15 × 10^3^, 5.08 × 10^4^, 5.15 × 10^5^, 5.08 × 10^6^, and 3.56 × 10^7^ PFU per animal for, respectively the 10^3^, 10^4^, 10^5^, 10^6^, or 10^7^ PFU group. No mice succumbed to disease or reached euthanasia criteria within the 21 days post challenge. A few mice in each group started losing body weight as early as Day 1 post infection (Figure 3A), with one subject of the 10^5^ PFU group losing 4.3% of its initial weight on Day 6, but weight loss did not progress for more than two consecutive days for any animals. Disease severity reached a maximum score of 3 for nearly half the cohort, but did not covary with the challenge dose (Figure 3B). Instead, the time at which mice showed first symptoms tended to be earlier with a higher dose. Specifically, five and one mouse from, respectively, the 10^7^ and 10^6^ PFU group, showed clinical signs of disease starting Day 6, whereas the first three, eight, and one mouse from, respectively, the 10^5^, 10^4^, or 10^3^ PFU group, showed signs one to two days later. Infectious virus was detected in all swabs from Day 2 to 6, except in those from three subjects of the lowest dose group on Day 6, with titers for most samples ranging between 10^2^ and 10^4^ PFU/mL (Figure 3C). Note that the lack of virus detection on Day 2 post infection in the susceptibility study compared to this study could be explained by a slight difference in the delivered dose for the 10^4^ PFU group, as well as the change in the limit of detection. Interestingly, while the average and distribution of the virus titers from the swabs of a given group were comparable between Day 2 and 6, the titers were significantly higher in animals challenged with the highest dose compared to the two lowest doses during the same time. Vaginal virus titers then dropped by Day 8, especially for those collected from animals in the three highest challenge dose groups, with titers being comparable between groups at that time (*p* > 0.05). Note that although sensitivity of detection was increased (LOD of 10 PFU/mL), no infectious virus was detected by Day 10 in any group and until the end of the study (Figure 3C). Interestingly, blood was isolated from vaginal swabs in four subjects at the start of Day 4 to 6, and up to Day 18 post infection for two subjects, but no correlation could be established with infective dose or clinical score. It is also unknown whether blood originated from a vaginal or uterine injury. Consistent with detection of the infectious virus in the vaginal cavity and subjects displaying mild signs of disease, most of them seroconverted to infection with a PRNT_50_ titer (reciprocal dilution) ranging between 10 and 60, regardless of the infective dose (Figure 3D). Note that one and two subjects in, respectively, the 10^5^ and 10^3^ PFU group, had a PRNT_50_ titer near 5 (or one not reliably calculable). Finally, none of the tissues (genital tract, liver, spleen, or kidney) collected from animals on Day 21 post challenge contained infectious virus. Altogether, these results were consistent with the first study, suggesting the susceptibility of mice to EBOV via the vaginal route, and the virus being significantly less virulent even when using a dose that is 3 log_10_ higher compared to the one delivered via IP route that caused 83% mortality in the same study.

## 4. Discussion

EBOV sexual transmission is poorly understood; it may be uncommon, or may simply have been underreported until recently. Indeed, despite the first isolation of Ebola virus in 1976 in the Democratic Republic of the Congo [5], there have been limited data about men-to-women sexual transmission, which was only documented for the first time during the 2014–2016 outbreak in Africa, which was the largest ever recorded [6,7]. Sexual transmission has also been suspected in several flare-up events in which multiple women succumbed to EVD [10,11,14,15]. Until recently, understanding EBOV sexual transmission in women was also impaired by the complete lack of experimental models of infection to examine the mechanisms and requirements of an effective transmission, and to evaluate antivirals. In a recent study [26], IFNAR^−/−^ mice, which are deficient in the type-I interferon receptor function, constituted the first lethal small animal model used to successfully model EBOV sexual transmission using wild-type and maEBOV isolates [26]. Here, we add to the field of characterizing animal models of filovirus sexual transmission by documenting the lack of virulence of an maEBOV isolate in wild-type BALB/c mice following an assisted intravaginal challenge.

The growth and differentiation of epithelial cells in the genital tract is tightly regulated by hormonal changes. To study sexually transmitted diseases in rodents, progesterone injection is a well-accepted procedure aiming to synchronize the reproductive cycle to the diestrus stage to thin the vaginal epithelium and increase the likelihood of infection [36,37,38]. Progesterone priming has also been reported to promote increased numbers of Langerhans cells in the vaginal epithelium [39], and since these are early target cells of EBOV [40], this could also be beneficial for model development. Specifically, priming mice with progesterone one week or less prior to challenge was previously shown to be required for herpes simplex virus to replicate and cause generalized mucocutaneous disease of the vagina [41,42,43], or to observe mortality using Zika virus [38]. A similar timeline for priming also allowed successful infection with *Chlamydia trachomatis* [44], and some mycoplasma species [45]. Here, we demonstrated that 3 mg Depo-Provera^®^ treatment of BALB/c mice six days prior maEBOV intravaginal challenge increased the number of mice that had virus replication in the vaginal vault, developed clinical signs of disease, and underwent seroconversion. However, none of the subjects exhibited more than a hunched posture or a loss of body weight, and none reached the euthanasia criteria. In contrast, BALB/c mice inoculated with a comparable challenge dose of virus through the I.P. route typically experienced severe disease with high lethality, as seen in our control group and in older studies [30,46,47]. Indeed, a mortality rate higher than 80% has been consistently achieved in this mouse model using MA-EBOV or its molecular clone rgMA-EBOV at 1000 focus-forming units (FFU) or less via the I.P. route, with a time to death typically ranging from 5 to 7 days and a calculated lethal dose (LD_50_) of 0.01 FFU [46,47]. Note that the equivalent titer from a focus assay (expressed in FFU) to a plaque assay (expressed in PFU) has been shown to be within 10-fold or less for filoviruses [48].

Performing progesterone priming at a closer time to challenge, as recently carried out in IFNAR^−/−^ mice [26] (2 days with 2 mg, instead of the 6 days with 3 mg in our study) is based on more recent studies on the sexual transmission of Zika virus in mice [37,38], and it is currently unknown whether this will impact our results and increase maEBOV virulence in BALB/c mice. Interestingly, a lower dose of Depo-Provera^®^ than what was used in our study has previously been shown to synchronize mice into a prolonged diestrus stage for more than 4 weeks [49], which encompasses the entire study length of 21 days, therefore suggesting that mice were adequately primed for the virus challenge. The quality of the inoculum is also unlikely to explain this effect, as the same virus stock was used to prepare the I.P. inoculum that provided the anticipated outcome. Besides, our data clearly indicate that animals seroconverted to infection via the vaginal route. Instead, the lack of maEBOV virulence following intravaginal challenge could stem from the fact that using peripheral routes for virus inoculation in this animal model does not typically cause disease, weight loss, or death. Indeed, a lack of clinical signs of disease and mortality has previously been documented when using subcutaneous, intramuscular, or intradermal inoculation with 100 PFU (equivalent to the 3000 LD_50_ in that study) or more, while still allowing seroconversion, which similar to our results, since animals were protected against an I.P. maEBOV challenge (100 PFU) performed three weeks later [30]. Conversely, the fact that severe disease and death was observed in IFNAR^−/−^ mice following both intravaginal [26] and subcutaneous [50] challenge using comparable EBOV doses rather suggests that the lack of type I interferon response renders these animals susceptible to peripheral inoculation routes; this appears to be more consistent with the disease severity seen in humans following sexual transmission.

Increased virulence or earlier onset of disease has been observed when using a higher infective dose and the I.P. route in BALB/c and other mouse models of infection [30,51,52], but again, no subjects succumbed to disease, lost significant body weight, or displayed more than a hunched posture in our serial dose study. However, our data are in line with these studies in the sense that clinical signs of disease appeared up to 2 days earlier, and virus titers from swabs were higher during the first 6 days when using the highest challenges doses. maEBOV-infected BALB/c mice typically display ruffled fur and hunched posture early in infection, followed by weight loss, lethargy, and moribundity [47], which we did not observe. Altogether, our data suggest that vaginal challenge results in subclinical infection regardless of the dose, which is supported by the quasi-uniform seroconversion of the 50 subjects, and is in line with previous studies using other peripheral routes of infection in the same animal model [30,50]. Our results also strikingly differ from the fact that IFNAR^−/−^ mice that survived wild-type and maEBOV vaginal challenges did not seroconvert by the end of the study [26].

To our knowledge, a challenge dose of 10^7^ PFU has never been reported in mice regardless of the route of inoculation. Uniform lethality has been consistently reached by using lower doses via the I.P. route, whereas 10^6^ PFU could not cause any noticeable disease by subcutaneous injection [26,30,47,51,52,53]. The viral load required to initiate vaginal mucosa infection in humans is unknown, and although a high inoculum is required to detect shedding of infectious virus over time in the human vaginal epithelium in vitro [29], a high dose such as 10^7^ PFU is unlikely to be biologically relevant. This overall reflects on the non-suitability of BALB/c mice to model typical EVD following vaginal challenge with maEBOV and for use as a model for the screening of microbicides; however, seroconversion could be used as a readout. Finally, changes in lethality rate have been noted when using maEBOV via the I.P. route in other laboratory wild-type mouse strains, including C57BL/6, which demonstrated increased survival with a higher dose [47]. However, testing doses lower than 10^3^ PFU will require further investigations.

Considering the fact that EBOV sexual transmission in humans can be fatal, future natural history of disease studies to characterize disease progression in BALB/c mice are not warranted at this time. However, the lack of virulence in this experimental set-up is of interest, and further investigations will be required to fully explain it.

## Data Availability

The data presented in this study are available on request from the corresponding authors.
